# Presentation and outcome in cancer patients with extensive spread to the brain

**DOI:** 10.1186/1756-0500-2-247

**Published:** 2009-12-12

**Authors:** Carsten Nieder, Adam Pawiniski, Astrid Dalhaug

**Affiliations:** 1 Department of Internal Medicine - Division of Oncology and Palliative Medicine, Nordland Hospital, 8092 Bodø, Norway; 2 Institute of Clinical Medicine, Faculty of Medicine, University of Tromsø, Tromsø, Norway

## Abstract

**Background:**

Controversy exists around the preferred management of patients with brain metastases and limited survival expectation, e.g. because of extensive brain involvement. Few studies have focused on this particular group of patients.

**Findings:**

A group of 24 patients with a large number of brain metastases, defined as 10 or more on computed tomography scans, who were managed with palliative whole-brain radiotherapy (WBRT), typically 30 Gy in 10 fractions, were analyzed. The median number of lesions was 14. The patient characteristics were comparable to those of studies in the general population with brain metastases, except for the fact that all patients had active sites of extracranial disease. Clinical benefit, imaging response and overall survival were lower than expected. Median survival, for example was 2 months. Trends towards better survival were found in patients with brain metastases detected at first cancer diagnosis (synchronous manifestation, treatment naïve) and those with better prognostic features according to the graded prognostic assessment (GPA) score.

**Conclusions:**

The benefit of WBRT did not meet the expectations, suggesting that consideration should be given to best supportive care including corticosteroid administration, especially if a patient belongs to the lowest GPA class.

## Findings

In patients with brain metastases, median overall survival typically is limited to 4-6 months after administration of palliative whole-brain radiotherapy (WBRT) [1]. It has long been recognised that patients with limited brain involvement, especially those with only one metastasis, represent a relatively favorable subgroup and that many of these patients are candidates for aggressive local treatment with surgery or radiosurgery, which aims at durable local control [2-10]. If the aim of local control can be achieved, long-term survival might be possible. The outcome of patients with extensive brain involvement is less well documented. Therefore, the purpose of the present study was to analyse the presentation and outcome in patients with a large number of brain metastases.

Since the opening of the Radiation Oncology facilities at the authors' institution early in 2007, all patients with brain metastases from solid tumors were entered into a database. All patients with 10 or more brain metastases on contrast-enhanced computed tomography (CT) scans and without clinical and radiological signs of carcinomatous meningitis were identified and retrospectively evaluated. Treatment consisted of WBRT (10 fractions of 3 Gy or 5 fractions of 4 Gy, individual steroid treatment as needed for symptom control, median dose 16 mg dexamethasone per day) without surgical resection or radiosurgery. We used the Kaplan-Meier method to generate actuarial survival curves. These were compared with the log rank test. Survival was calculated from the first day of treatment. All patients had died at the time of analysis. A p-value < 0.05 was considered statistically significant.

The study group included 24 patients with 10 or more brain metastases on CT scans (median 14, maximum approximately 50). Two patients failed to complete their prescribed treatment (continuous clinical deterioration), but are included in this analysis. All patients had known sites of extracranial disease, either uncontrolled primary tumor or metastases. Table 1 shows the patient characteristics. The maximum diameter of the largest brain lesion was more than 3 cm in 4 patients, 2-3 cm in 8 patients, 1-2 cm in 9 patients and less than 1 cm in 3 patients (17%, 33%, 37.5% and 12.5%, respectively).

**Table 1:** Patient characteristics

Median age, range	56 yrs., 36-80
Median KPS, range	70, 30-90

Metachronous vs. synchronous brain metastases	67 vs. 33% (16 vs. 8)

Without extracranial metastases	21% (5)

RPA class I vs. II vs. III	0:50:50% (0:12:12)

GPA class I vs. II vs. III vs. IV	0:0:37.5:62.5% (0:0:15:9)

Male vs. female gender	62.5 vs. 37.5% (15 vs. 9)

Non-small cell lung cancer	25% (6)

Small-cell lung cancer	25% (6)

Breast cancer	21% (5)

Malignant melanoma	17% (4)

Prostate cancer	4% (1)

Rectal cancer	4% (1)

Uterine cancer	4% (1)

KPS: Karnofsky performance status, RPA: recursive partitioning analysis [11], GPA: graded prognostic assessment [12]

**Figure 1:**
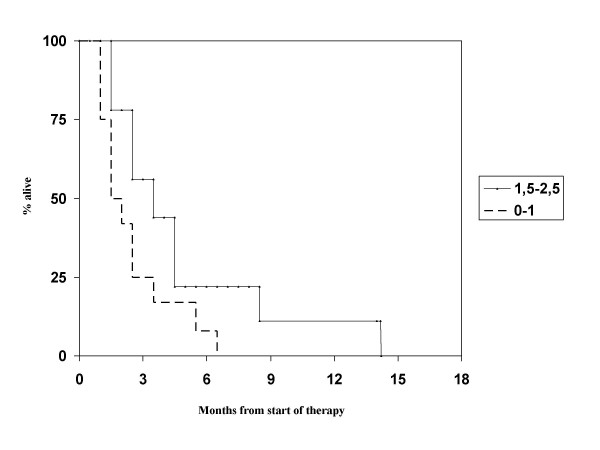
**Kaplan-Meier estimates of overall survival in 24 patients with at least 10 brain metastases grouped by graded prognostic assessment (GPA) score, p > 0.1**. In the GPA system, the most unfavorable group has 0-1 points, the two intermediate groups have 1.5-2.5 and 3 points, respectively. The most favorable group has 3.5-4 points. None of the present patients had more than 2.5 points.

The clinical benefit was assessed by the treating physician in 21 patients (improvement of symptoms and/or performance status). No information was available for 3 patients who immediately returned to their local hospitals. Six patients (25%) were judged to have experienced clinical improvement. Five had continuous deterioration (21%), the other 10 were stable (42%). Follow-up CT was scheduled approximately 5-6 weeks and 3 months after WBRT. However, 7 patients were never assessed with repeated CT imaging. In the remainder 17, 8 (33%) had partial responses (decrease of all lesions by at least 50% without appearance of new lesions) on at least one CT examination. No complete remission was seen. Five of the 8 radiological responders also had clinical improvement. In 6 patients with small cell lung cancer, two clinical and one imaging response were seen. Median overall survival was 2 months. At 6 months, 3 out of 24 patients (12.5%) were alive. The maximum survival was 14 months. For the recursive partitioning analysis (RPA) class II (Karnofsky performance status (KPS) at least 70%, but at least one adverse diagnostic feature such as age ≥65 years, uncontrolled primary tumor or extracranial metastases) [11], median overall survival was 2.0 months and maximum survival 14 months (in a patient with small cell lung cancer). For RPA class III, defined by KPS < 70, median overall survival was 1.5 months and maximum survival 6 months (in a patient with breast cancer). Figure 1 shows overall survival according to the new graded prognostic assessment (GPA) score [12], which performed better than the RPA classes even if no significant p-value was calculated with the available number of patients. The 4 GPA classes are based on a score where 0, 0.5 or 1 point is assigned for age, number of brain metastases, absence of extracranial metastases and KPS. We were unable to identify any factor significantly associated with better survival or likelihood of clinical benefit. A trend was seen for better survival in patients with synchronous manifestation of brain metastases and those with better GPA score (p > 0.1).

This is to the authors' best knowledge the second report that specifically addresses the outcome of patients with a large number of multiple brain metastases, arbitrarily defined as 10 or more on CT scans. One has to assume that magnetic resonance imaging (MRI) would detect an even higher number of lesions in these patients (possibly some cases with leptomeningeal metastases too), but there was no reason to perform MRI as this would not have altered the patients' management. The cohort we identified is relatively small and this fact should be taken into account when interpreting the results, e.g., with regard to power of statistical tests.

Despite possible limitations of small retrospective studies, it is intriguing to see that all patients had active extracranial disease (metastases and/or uncontrolled primary tumor), thus none of them could be grouped into RPA class I. Apart from this, patient characteristics were unremarkable compared to other series. Overall survival in RPA class III was at the lower end of the range of previously reported results [13-15]. However, class II had lower median survival than the often quoted 3.5-4 months [11,13-16]. This might result from the unusually advanced brain involvement, which apparently is associated with lower than expected rates of clinical and imaging response after typical palliative WBRT regimens. Also for the complete group of patients, median overall survival (2 months) was poorer than expected. The GPA score appears to predict the survival of our patients quite well, yet no data are available for the favorable GPA classes.

Our survival data raise the question of whether these patients can be managed with best supportive care including steroids and anticonvulsants rather than WBRT plus these measures. Head to head comparisons of the two strategies are not available, but a large series that included 118 patients managed with steroids without WBRT [17] arrived at survival figures at 2 and 3 months which are overlapping with ours. Some patients in the study actually survived for 6 months and more [17]. No data on neurologic function, performance status or quality of life is yet available, although such endpoints might be more important than slight differences in survival. The currently active randomised Medical Research Council QUARTZ trial comparing optimal supportive care and steroids with or without WBRT in patients with non-small cell lung cancer will contribute important information on the management of patients with brain metastases and poor prognosis [18]. Omission of WBRT might be considered especially in patients with active extracranial disease where no further systemic treatment options exist and where the extracranial tumor load is as threatening as the brain involvement.

If WBRT seems indicated, different dose-/fractionation regimens can be chosen. In patients with more than 3 brain metastases, the outcome was similar with WBRT with 5 fractions of 4 Gy and 10 fractions of 3 Gy in the series by Rades et al. [19]. The present results also indicate that short course radiotherapy is preferable. For the occasional patient with stable or responding extracranial disease, local control of the brain metastases might eventually impact on survival. Several groups even offered radiosurgery to patients with a large number of lesions because the long-term local control probability after WBRT alone is known to be inferior to that of radiosurgery [1]. The study by Bhatnagar et al. suggests that patients with 7-18 brain metastases might survive for a median of 6 months after upfront or salvage radiosurgery, but no data for the subgroup with 10 or more metastases are available [20]. One should also be aware of the fact that radiosurgery-treated patients are different from those reported here, e.g. with regard to imaging with MRI with high doses of Gd-contrast media, smaller brain lesions and often less aggressive extracranial disease. Own data suggest that WBRT will control most of the small lesions for the remaining life time [21,22] and therefore one might prefer to administer radiosurgery boost to a limited number of targets and select these according to size and location, taking into account whether or not a lesion is likely to contribute to neurological deficits and what the risk of radiation toxicity will be. Amendola et al. decided to administer radiosurgery without WBRT in 72 patients with 10 or more lesions, but on average used 2 treatment sessions (to the authors' knowledge the first paper specifically addressing patients with 10 or more metastases) [23]. Their patients had lesions detected by MRI with triple-dose Gd-contrast enhancement. The exact size distribution was not reported. Nevertheless one has to assume that brain involvement in our own patient group was more extensive as it was depicted on CT scans. In both series, all patients had extracranial disease. Median age was comparable. Whether the radiosurgery study included patients with small cell lung cancer was not reported. Median survival after radiosurgery was 4.3 months and 6-months survival approximately 37% (estimated from the published graphs), i.e. better than in our series but inferior to typical radiosurgery series in patients with few brain metastases. The contribution of selection bias to these results is difficult to estimate. In conclusion, the typical patient with estimated survival of approximately 2 months has two options: best supportive care or additional short course WBRT. Prospective data on neurologic function, symptom load and quality of life need to be collected to support decision making in a situation where aggressive oncological treatment no longer is appropriate.

## Competing interests

The authors declare that they have no competing interests.

## Authors' contributions

CN, AP and AD participated in the design of the study, CN and AP collected patient data and follow-up information, CN carried out the statistical analysis, CN, AP and AD drafted the manuscript. All authors read and approved the final manuscript.
